# Supplementary material for: Near-Infrared Spectroscopy (NIRS) as a Tool for Classification of Pre-Sliced Iberian Salchichón, Modified Atmosphere Packaged (MAP) According to the Official Commercial Categories of Raw Meat
Source: Foods. 2021 Aug 12;10(8):1865. doi: 10.3390/foods10081865 (PMC8393770; doi:10.3390/foods10081865)
Supplement: Supplementary file 1 [file foods-10-01865-s001.zip › foods-1248306-supplementary.pdf]

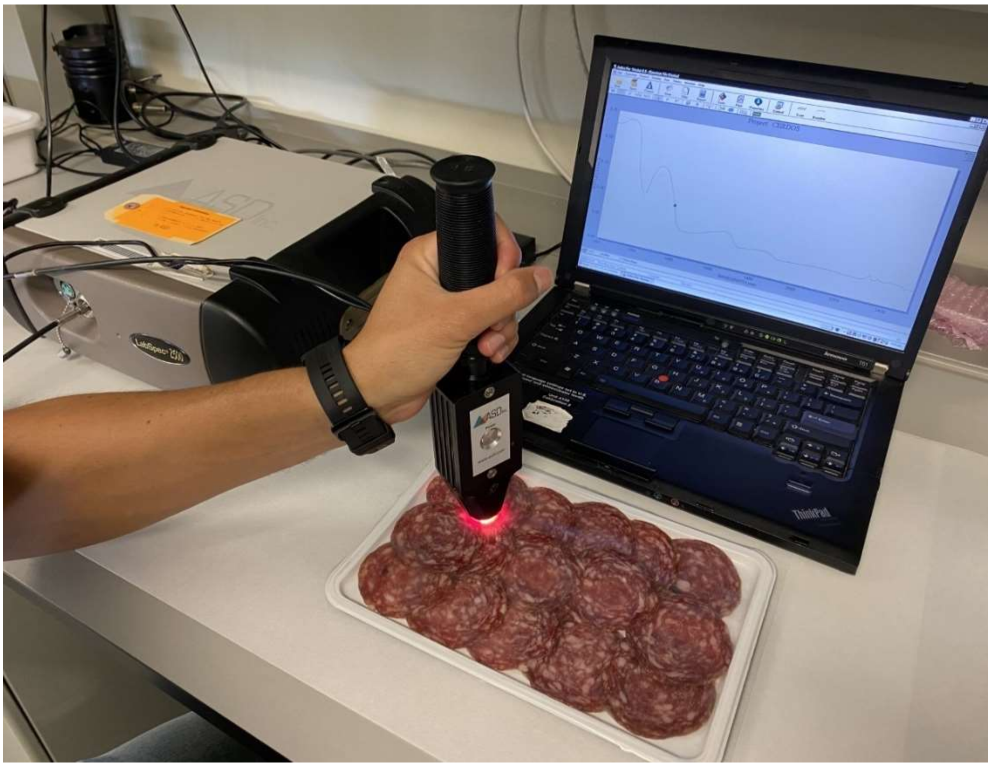

Figure S1. Spectral sampling (reflectance) of a sample of Iberian *salchichón* under modified atmosphere packaging with a LabSpec 2500 (ASD Inc., USA) NIRS spectrometer equipped with an ASD fi-bre-optic contact Probe® (21-mm window diameter).

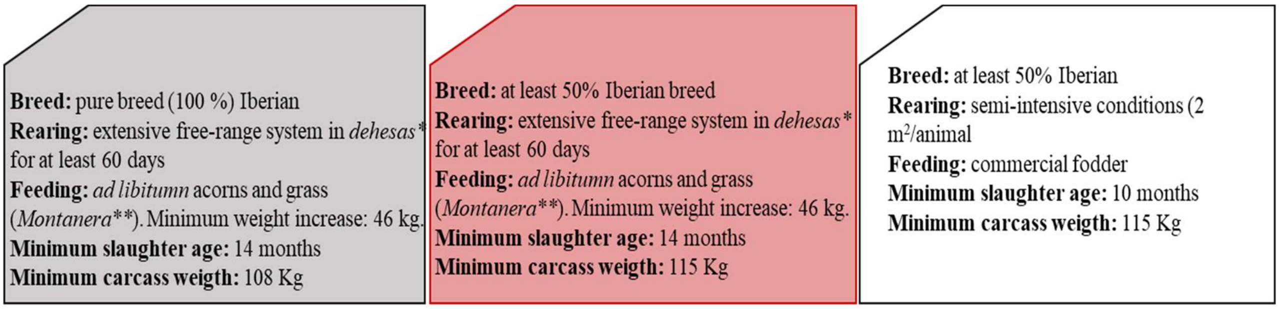

Figure S2. Production system conditions required in *Black, Red* and *White* commercial category according to the current Spanish Iberian Quality Standard. **dehesas*: rangelands with evergreen oaks and pastures that are found in the southwest of the Iberian Peninsula used for extensive livestock farming with mixed-species grazing (beef cattle, sheep, and Iberian pigs). **Montanera*, typical free-range system from South-West Iberian Peninsula with feeding mainly based on acorn and grass.

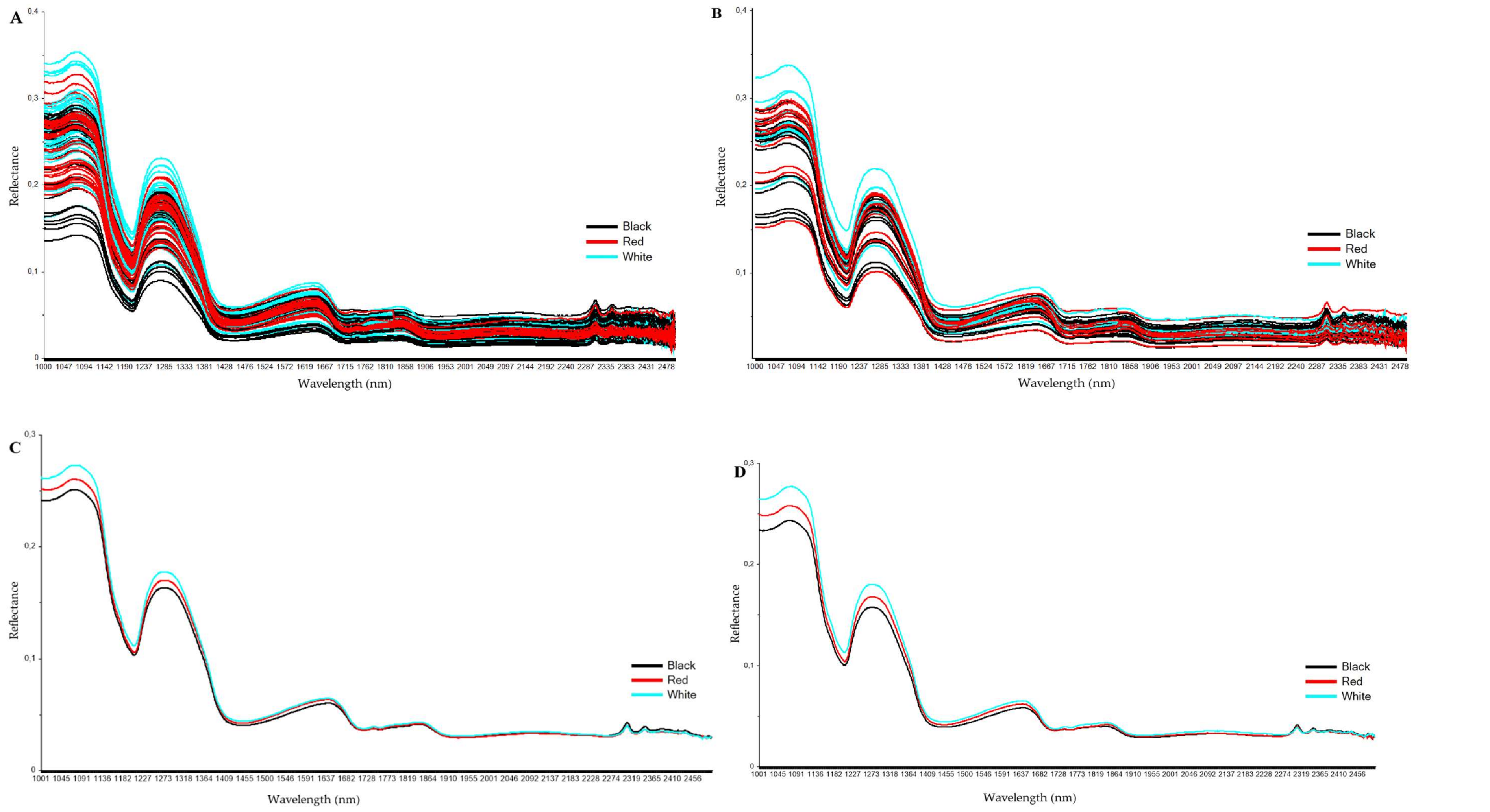

Figure S3. Raw spectra (reflectance) of total of samples (A) and mean spectra (C) of calibration and total of samples (B) and mean spectra (D) of external validation sample sets of pre-sliced MAP packages of Iberian *salchichón* (1000-2500 nm) grouped by commercial category of the raw material (*Black, Red* and *White*) used for its manufacture.

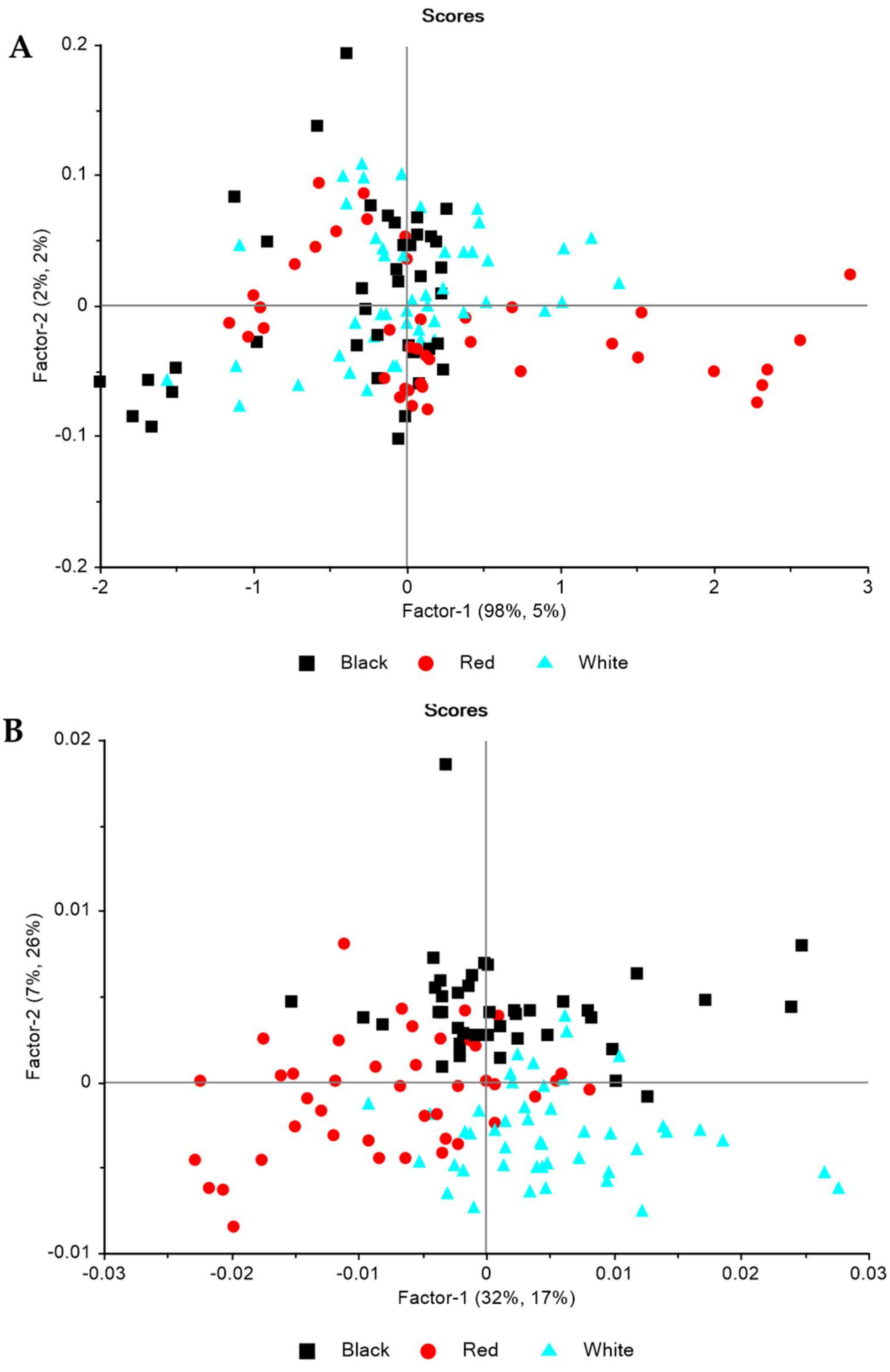

Figure S4: 2-D scatter PCA analysis plot of pre-sliced modified atmosphere packaged Iberian *salchichón* samples in (A) Absorbance at 1000-1800 nm and after (B) SNV-DE SG 1,4,4,1 (Absorbance) at 1000-1800 nm. Samples were grouped by official commercial categories (*Black*, *Red* and *White*) of raw material used for manufacturing Iberian *salchichón.* Graphical representation of PC1 (98%, 5%) vs PC2 (2%, 2%) for A and PC1 (32%, 17%) vs PC2 (7%, 26%) for B, for calibration and cross-validation, respectively.

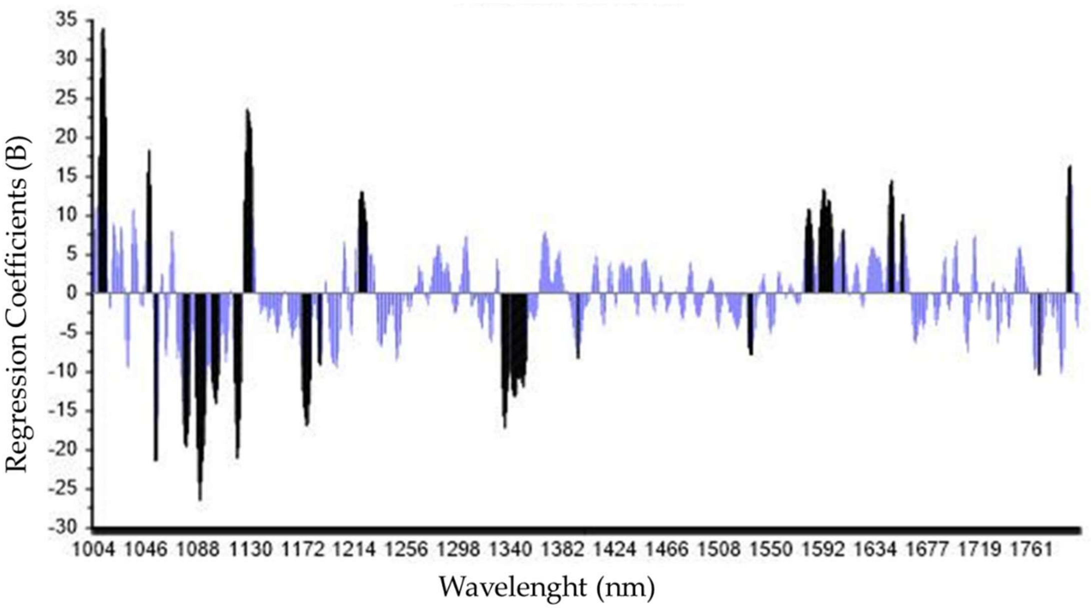

Figure S5: PLS-DA analysis after SNV-DE SG 1,4,4,1 (Absorbance (log1/R)) at 1000-1800 nm: graphical representation of regression coefficients (B) of wavelengths of Iberian *salchichón* spectral data. Significant variables are highlighted in black.

Table S1. PLS-DA results of pre-sliced MAP packaged Iberian *salchichón* within the official commercial categories of the raw material (*Black, Red* and *White*) according to various spectral pre-treatments.

Pre-treatment	LVs	n	Calibration												External validation						
			Black				*Red*				*White*				n	*Black*		*Red*		*White*	
			1-VR	RMESECV	SE	SP	1-VR	RMESECV	SE	SP	1-VR	RMESECV	SE	SP		SE	SP	SE	SP	SE	SP
Absorbance	12	127	0.498	0.329	46.51	72.63	0.552	0.313	53.49	74.73	0.681	0.276	77.08	76.67	47	37.50	38.71	25.00	67.74	53.33	65.63
SNV-DE	12	130	0.589	0.296	46.51	73.68	0.617	0.295	63.83	65.93	0.737	0.248	85.42	80.00	47	31.25	41.94	50.00	61.29	46.67	71.88
SG 1,4,4,1	9	129	0.580	0.298	65.12	75.79	0.615	0.297	75.59	80.22	0.789	0.223	87.50	96.67	47	43.75	48.39	37.50	67.74	60.00	78.13
SNV-DE SG 1,4,4,1	8	128	**0.599**	**0.296**	**69.77**	**81.05**	**0.653**	**0.279**	**78.72**	**80.22**	**0.782**	**0.226**	**91.67**	**92.22**	47	**43.75**	**45.16**	**43.75**	**70.97**	**46.67**	**78.13**
SG 2,5,5,2	4	131	0.390	0.360	32.56	64.21	0.476	0.346	44.68	57.14	0.569	0.317	58.33	77.78	47	12.50	45.16	12.50	45.16	31.25	56.25
SNV-DE SG 2,5,5,2	4	128	0.480	0.337	53.49	76.84	0.456	0.351	55.32	61.54	0.562	0.319	54.17	73.33	47	31.25	51.61	18.75	51.61	40.00	65.63

Black, Red and *White* = commercial categories of raw material defined by the current Spanish Iberian Quality Standard used for manufacturing Iberian *salchichón*; SNV = Standard normal variate; DE = de-trending; SG = Savitzky-Golay derivates; LVs = Latent variables; n = Number of samples; 1-VR = Coefficient of determination in cross-validation; RMSECV = Root mean square error of cross validation; SE = Sensitivity; SP = Specificity.

Table S2. SIMCA results of pre-sliced MAP packaged Iberian *salchichón* within the official commercial categories of the raw material (*Black, Red* and *White*) according to various spectral pre-treatments.

		Calibration										External validation					
Pre-treatment	n	*Black*			*Red*			*White*			n	*Black*		*Red*		*White*	
		PCs	SE	SP	PCs	SE	SP	PCs	SE	SP		SE	SP	SE	SP	SE	SP
Abs	138	2	**100.00**	**18.95**	1	**95.74**	**3.30**	2	**100.00**	**23.33**	47	**100.00**	**19.35**	**87.50**	**3.22**	**100.00**	**21.88**
SNV-DE	138	7	100.00	14.74	7	97.87	7.69	7	100.00	18.68	47	93.75	22.58	100.00	3.22	100.00	15.63
SG 1,4,4,1	138	2	100.00	14.74	2	100.00	0.00	3	100.00	17.78	47	100.00	22.58	93.75	0.00	93.33	18.75
SNV-DE SG 1,4,4,1	138	7	97.67	0.00	6	100.00	4.40	5	100.00	5.56	47	100.00	3.22	100.00	6.45	100.00	6.25
SG 2,5,5,2	138	2	97.67	10.99	1	100.00	0.00	4	97.92	7.78	47	100.00	3.22	100.00	0.00	100.00	3.12
SNV-DE SG 2,5,5,2	138	6	90.70	0.00	7	91.49	0.00	7	93.75	0.00	47	100.00	0.00	100.00	0.00	100.00	0.00

Black, Red and *White* = commercial categories of raw material defined by the current Spanish Iberian Quality Standard used for manufacturing Iberian s*alchichón*; SNV = Standard normal variate; DE = de-trending; SG = Savitzky-Golay derivates; PCs = Number of principal components; n = Number of samples; SE = Sensitivity; SP = Specificity.

Table S3. LDA results of pre-sliced MAP packaged Iberian *salchichón* within the official commercial categories of the raw material (*Black, Red* and *White*) according to various spectral pre-treatments.

		Calibration							External validation					
Pre-treatment	n	*Black*		*Red*		*White*		n	*Black*		*Red*		*White*	
		SE	SP	SE	SP	SE	SP		SE	SP	SE	SP	SE	SP
Abs	138	60.47	54.74	38.29	25.27	14.58	31.11	47	68.75	9.68	50.00	25.81	26.67	37.50
SNV-DE	138	**81.40**	**91.58**	**87.23**	**87.91**	**79.17**	**94.44**	47	**75.00**	**80.65**	**81.25**	**77.42**	**53.33**	**96.88**
SG 1,4,4,1	138	37.21	81.32	66.00	62.64	56.25	85.56	47	43.75	83.87	56.25	70.97	46.67	68.75
SNV-DE SG 1,4,4,1	138	86.05	74.74	74.47	89.01	62.50	97.78	47	81.25	74.19	75.00	93.55	60.00	90.63
SG 2,5,5,2	138	27.91	70.51	55.32	62.64	43.75	81.11	47	43.75	70.97	62.50	64.52	33.33	84.38
SNV-DE SG 2,5,5,2	138	55.81	71.58	63.83	80.22	52.08	84.44	47	56.25	74.19	68.75	77.42	53.33	87.50

Black, Red and *White* = commercial categories of raw material defined by the current Spanish Iberian Quality Standard used for manufacturing Iberian *salchichón*; SNV = Standard normal variate; DE = de-trending; SG = Savitzky-Golay derivates; n = Number of samples; SE = Sensitivity; SP = Specificity.

Table S4. NIRS predictive results for dry matter (g/100g) in pre-sliced MAP Iberian *salchichón* according to various spectral pre-treatments.

Pre-treatment	LVs	n	Calibration		External Validation	
			1-RV	RMSECV	R^2_v	RMSEV
Absorbance	11	121	0.659	0.691	0.242	1.204
SNV-DE	10	122	**0.704**	**0.700**	**0.204**	**1.234**
SG 1,4,4,1	3	121	0.517	0.785	0.447	1.030
SNV-DE SG 1,4,4,1	2	120	0.553	0.804	0.305	1.154
SG 2,5,5,2	2	138	0.342	1.083	0.307	1.152
SNV-DE SG 2,5,5,2	1	138	0.407	1.028	0.244	1.203

SNV = Standard normal variate; DE = de-trending; SG = Savitzky-Golay derivates; LVs = Latent variables; n = Number of samples; 1-VR = Coefficient of determination in cross-validation; RMSECV = Root mean square error of cross validation; R^2_v = The determination coefficient of external validation; RMSEV = Root mean square error of validation.

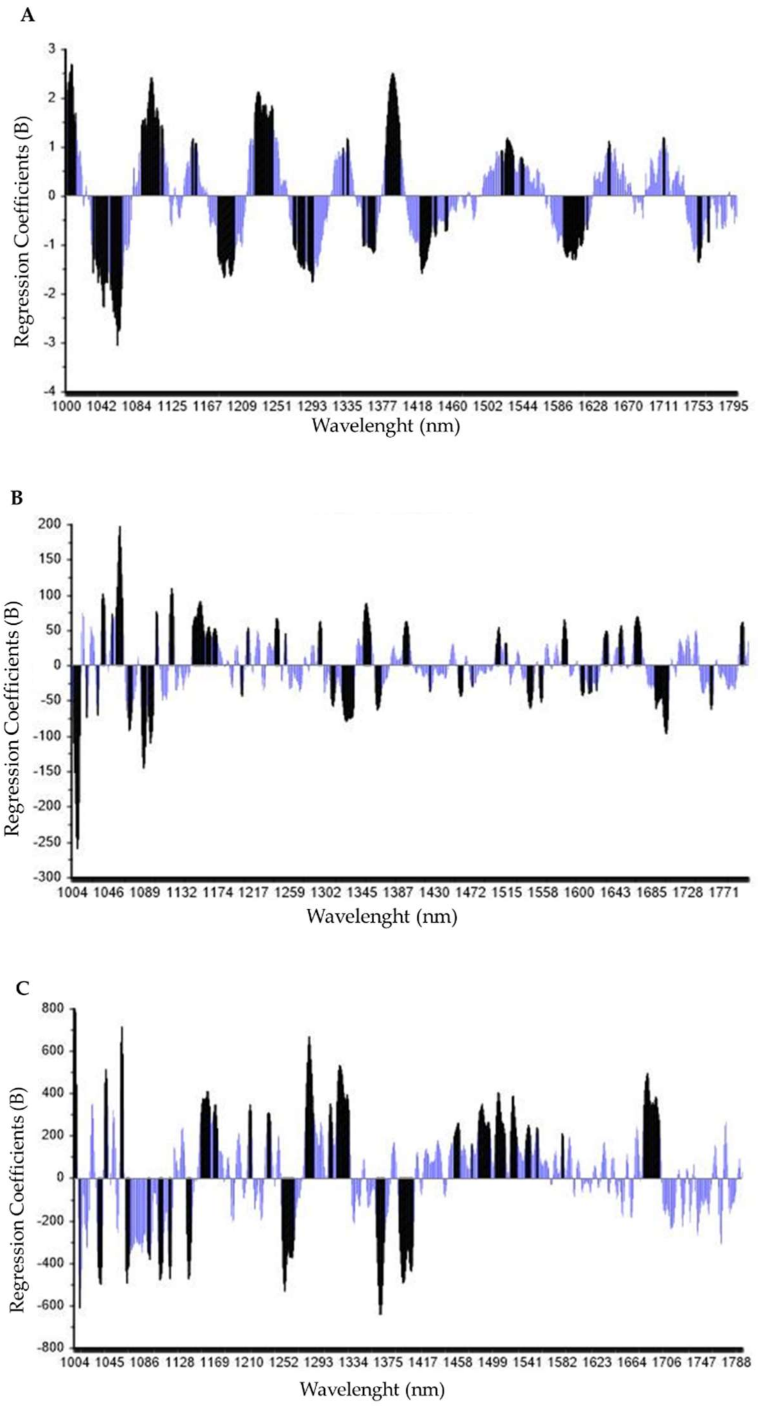

Figure S6: PLS analysis after SNV-DE (A), SNV-DE SG 1,4,4,1 (B) and SG 1,4,4,1 (C) of the Absorbance (log1/R)) at 1000-1800 nm: graphical representation of regression coefficients (B) of wavelengths of Iberian *salchichón* spectral data. Significant variables are highlighted in black.

Table S5. NIRS predictive results for NaCl (g/100g) in pre-sliced MAP Iberian *salchichón* according to various spectral pre-treatments.

Pre-treatment	LVs	n	Calibration		External Validation	
			1-RV	RMSECV	R^2v	RMSEV
Absorbance	12	124	0.502	0.205	0.386	0.247
SNV-DE	10	123	**0.687**	**0.715**	**NA**	**69.439**
SG 1,4,4,1	3	138	0.443	0.997	NA	69.539
SNV-DE SG 1,4,4,1	6	120	0.588	0.189	0.424	0.239
SG 2,5,5,2	2	126	0.371	0.231	0.364	0.251
SNV-DE SG 2,5,5,2	2	122	0.416	0.214	0.463	0.231

SNV = Standard normal variate; DE = de-trending; SG = Savitzky-Golay derivates; LVs = Latent variables; n = Number of samples; 1-VR = Coefficient of determination in cross-validation; RMSECV = Root mean square error of cross validation; R^2v = The determination coefficient of external validation; RMSEV = Root mean square error of validation.

Table S6. NIRS predictive results for α-tocopherol (µg/g) in pre-sliced MAP Iberian *salchichón* according to various spectral pre-treatments.

Pre-treatment	LVs	n	Calibration		External Validation	
			1-RV	RMSECV	R^2_V	RMSEV
Absorbance	12	119	0.680	1.742	0.422	2.441
SNV-DE	11	120	0.645	1.771	0.506	2.258
SG 1,4,4,1	6	116	0.728	1.434	0.511	2.245
SNV-DE SG 1,4,4,1	5	117	**0.730**	**1.522**	**0.601**	**2.029**
SG 2,5,5,2	2	120	0.496	1.986	0.367	2.556
SNV-DE SG 2,5,5,2	3	115	0.607	1.785	0.400	2.487

SNV = Standard normal variate; DE = de-trending; SG = Savitzky-Golay derivates; LVs = Latent variables; n = Number of samples; 1-VR = Coefficient of determination in cross-validation; RMSECV = Root mean square error of cross validation; R^2_V = The determination coefficient of external validation; RMSEV = Root mean square error of validation.

Table S7. NIRS predictive results for γ-tocopherol (μg/g) in pre-sliced MAP Iberian *salchichón* according to various spectral pre-treatments.

Pre-treatment	LVs	n	Calibration		External Validation	
			1-RV	RMSECV	R^2V	RMSEV
Absorbance	4	127	0.125	0.178	0.163	0.236
SNV-DE	9	128	0.398	0.149	0.312	0.214
SG 1,4,4,1	4	122	0.397	0.131	0.358	0.207
SNV-DE SG 1,4,4,1	5	125	**0.731**	**1.633**	**NA**	**9.454**
SG 2,5,5,2	2	127	0.272	0.147	0.136	0.240
SNV-DE SG 2,5,5,2	1	138	0.202	0.209	0.175	0.234

SNV = Standard normal variate; DE = de-trending; SG = Savitzky-Golay derivates; LVs = Latent variables; n = Number of samples; 1-VR = Coefficient of determination in cross-validation; RMSECV = Root mean square error of cross validation; R^2v = The determination coefficient of external validation; RMSEV = Root mean square error of validation.

Table S8. NIRS predictive results for palmitic acid (g/100g fatty acid methyl esters) in pre-sliced MAP Iberian *salchichón* according to various spectral pre-treatments.

Pre-treatment	LVs	n	Calibration		External Validation	
			1-RV	RMSECV	R^2_V	RMSEV
Absorbance	12	127	0.547	0.593	0.033	0.794
SNV-DE	9	130	0.477	0.594	0.373	0.640
SG 1,4,4,1	6	128	0.634	0.539	0.231	0.716
SNV-DE SG 1,4,4,1	6	128	**0.651**	**0.560**	**0.184**	**0.729**
SG 2,5,5,2	4	127	0.341	0.649	0.091	0.770
SNV-DE SG 2,5,5,2	1	138	0.294	0.818	0.111	0.761

SNV = Standard normal variate; DE = de-trending; SG = Savitzky-Golay derivates; LVs = Latent variables; n = Number of samples; 1-VR = Coefficient of determination in cross-validation; RMSECV = Root mean square error of cross validation; R^2_V = The determination coefficient of external validation; RMSEV = Root mean square error of validation.

Table S9. NIRS predictive results for stearic acid (g/100g fatty acid methyl esters) in pre-sliced MAP Iberian *salchichón* according to various spectral pre-treatments.

Pre-treatment	LVs	n	Calibration		External Validation	
			1-RV	RMSECV	R^2_V	RMSEV
Absorbance	8	129	0.379	0.738	0.479	0.728
SNV-DE	8	120	0.544	0.468	0.498	0.715
SG 1,4,4,1	6	124	0.581	0.639	0.427	0.763
SNV-DE SG 1,4,4,1	6	128	**0.728**	**0.597**	**0.554**	**0.673**
SG 2,5,5,2	5	127	0.279	0.928	0.190	0.908
SNV-DE SG 2,5,5,2	1	138	0.269	1.176	0.260	0.868

SNV = Standard normal variate; DE = de-trending; SG = Savitzky-Golay derivates; LVs = Latent variables; n = Number of samples; 1-VR = Coefficient of determination in cross-validation; RMSECV = Root mean square error of cross validation; R^2_V = The determination coefficient of external validation; RMSEV = Root mean square error of validation.

Table S10. NIRS predictive results for oleic acid (g/100g fatty acid methyl esters) in pre-sliced MAP Iberian *salchichón* according to various spectral pre-treatments.

Pre-treatment	LVs	n	Calibration		External Validation	
			1-RV	RMSECV	R^2v	RMSEV
Absorbance	12	123	0.423	0.928	0.176	1.001
SNV-DE	9	128	0.504	0.966	0.465	0.807
SG 1,4,4,1	6	126	**0.612**	**0.889**	**0.118**	**1.036**
SNV-DE SG 1,4,4,1	5	124	0.569	0.759	0.337	0.898
SG 2,5,5,2	2	138	0.191	1.494	NA	1.108
SNV-DE SG 2,5,5,2	2	125	0.383	0.975	NA	1.112

SNV = Standard normal variate; DE = de-trending; SG = Savitzky-Golay derivates; LVs = Latent variables; n = Number of samples; 1-VR = Coefficient of determination in cross-validation; RMSECV = Root mean square error of cross validation; R^2v = The determination coefficient of external validation; RMSEV = Root mean square error of validation.

Table S11. NIRS predictive results for linoleic acid (g/100g fatty acid methyl esters) in pre-sliced MAP Iberian s*alchichón* according to various spectral pre-treatments.

Pre-treatment	LVs	n	Calibration		External Validation	
			1-RV	RMSECV	R^2_V	RMSEV
Absorbance	10	128	0.607	0.208	0.487	0.237
SNV-DE	7	128	0.584	0.222	0.511	0.231
SG 1,4,4,1	6	129	**0.652**	**0.206**	**0.386**	**0.259**
SNV-DE SG 1,4,4,1	4	128	0.634	0.209	0.429	0.250
SG 2,5,5,2	5	122	0.545	0.217	0.073	0.318
SNV-DE SG 2,5,5,2	1	125	0.336	0.265	0.303	0.276

SNV = Standard normal variate; DE = de-trending; SG = Savitzky-Golay derivates; LVs = Latent variables; n = Number of samples; 1-VR = Coefficient of determination in cross-validation; RMSECV = Root mean square error of cross validation; R^2_V = The determination coefficient of external validation; RMSEV = Root mean square error of validation.

Table S12. NIRS predictive results for linolenic acid (g/100g fatty acid methyl esters) in pre-sliced MAP Iberian s*alchichón* according to various spectral pre-treatments.

Pre-treatment	LVs	n	Calibration		External Validation	
			1-RV	RMSECV	R^2v	RMSEV
Absorbance	10	130	0.725	0.159	0.644	0.194
SNV-DE	12	133	0.794	0.155	0.719	0.172
SG 1,4,4,1	5	133	0.789	0.160	0.724	0.170
SNV-DE SG 1,4,4,1	4	134	**0.824**	**0.146**	**0.808**	**0.142**
SG 2,5,5,2	3	125	0.698	0.161	0.535	0.221
SNV-DE SG 2,5,5,2	3	125	0.748	0.151	0.512	0.227

SNV = Standard normal variate; DE = de-trending; SG = Savitzky-Golay derivates; LVs = Latent variables; n = Number of samples; 1-VR = Coefficient of determination in cross-validation; RMSECV = Root mean square error of cross validation; R^2v = The determination coefficient of external validation; RMSEV = Root mean square error of validation.

Table S13. NIRS predictive results for lipid oxidation index (mg MDA/kg) in pre-sliced MAP Iberian *salchichón* according to various spectral pre-treatments.

Pre-treatment	LVs	n	Calibration		External Validation	
			1-RV	RMSECV	R^2_V	RMSEV
Absorbance	12	125	0.639	0.252	0.417	0.318
SNV-DE	12	127	0.680	0.241	0.355	0.335
SG 1,4,4,1	6	125	0.687	0.235	0.513	0.291
SNV-DE SG 1,4,4,1	8	124	**0.746**	**0.213**	**0.342**	**0.338**
SG 2,5,5,2	3	125	0.462	0.302	0.350	0.336
SNV-DE SG 2,5,5,2	2	123	0.556	0.270	0.342	0.338

SNV = Standard normal variate; DE = de-trending; SG = Savitzky-Golay derivates; LVs = Latent variables; n = Number of samples; 1-VR = Coefficient of determination in cross-validation; RMSECV = Root mean square error of cross validation; R^2_V = The determination coefficient of external validation; RMSEV = Root mean square error of validation.

Table S14. NIRS predictive results for protein oxidation index (nmol carbonyls/mg protein) in pre-sliced MAP Iberian *salchichón* according to various spectral pre-treatments.

Pre-treatment	LVs	n	Calibration		External Validation	
			1-RV	RMSECV	R^2_V	RMSEV
Absorbance	5	128	0.214	0.406	NA	0.523
SNV-DE	10	130	0.334	0.400	NA	0.536
SG 1,4,4,1	6	130	0.394	0.389	0.115	0.486
SNV-DE SG 1,4,4,1	4	126	**0.441**	**0.343**	**0.065**	**0.499**
SG 2,5,5,2	2	124	0.295	0.385	0.106	0.488
SNV-DE SG 2,5,5,2	1	133	0.228	0.425	0.103	0.489

SNV = Standard normal variate; DE = de-trending; SG = Savitzky-Golay derivates; LVs = Latent variables; n = Number of samples; 1-VR = Coefficient of determination in cross-validation; RMSECV = Root mean square error of cross validation; R^2_V = The determination coefficient of external validation; RMSEV = Root mean square error of validation.
